# Reproduction, Smell, and Neurodevelopmental Disorders: Genetic Defects in Different Hypogonadotropic Hypogonadal Syndromes

**DOI:** 10.3389/fendo.2014.00109

**Published:** 2014-07-09

**Authors:** Hernan Valdes-Socin, Matilde Rubio Almanza, Mariana Tomé Fernández-Ladreda, François Guillaume Debray, Vincent Bours, Albert Beckers

**Affiliations:** ^1^Service of Endocrinology, CHU Liège, University of Liège, Liège, Belgium; ^2^Service of Human Genetics, CHU Liège, University of Liège, Liège, Belgium

**Keywords:** reproduction, male, Kallman syndrome, hypogonadotropic hypogonadism, olfaction, kisspeptin, genetics

## Abstract

The neuroendocrine control of reproduction in mammals is governed by a neural hypothalamic network of nearly 1500 gonadotropin-releasing hormone (GnRH) secreting neurons that modulate the activity of the reproductive axis across life. Congenital hypogonadotropic hypogonadism (HH) is a clinical syndrome that is characterized by partial or complete pubertal failure. HH may result from inadequate hypothalamic GnRH axis activation, or a failure of pituitary gonadotropin secretion/effects. In man, several genes that participate in olfactory and GnRH neuronal migration are thought to interact during the embryonic life. A growing number of mutations in different genes are responsible for congenital HH. Based on the presence or absence of olfaction dysfunction, HH is divided in two syndromes: HH with olfactory alterations [Kallmann syndrome (KS)] and idiopathic hypogonadotropic hypogonadism (IHH) with normal smell (normosmic IHH). KS is a heterogeneous disorder affecting 1 in 5000 males, with a three to fivefold of males over females. KS is associated with mutations in *KAL1, FGFR1/FGF8, FGF17, IL17RD, PROK2/PROKR2, NELF, CHD7, HS6ST1, FLRT3, SPRY4, DUSP6, SEMA3A, NELF*, and *WDR11* genes that are related to defects in neuronal migration. These reproductive and olfactory deficits include a variable non-reproductive phenotype, including sensorineural deafness, coloboma, bimanual synkinesis, craniofacial abnormalities, and/or renal agenesis. Interestingly, defects in *PROKR2, FGFR1, FGF8, CHD7, DUSP6*, and *WDR11* genes are also associated with normosmic IHH, whereas mutations in *KISS1/KISSR, TAC3/TACR3, GNRH1/GNRHR, LEP/LEPR, HESX1, FSHB*, and *LHB* are only present in patients with normosmic IHH. In this paper, we summarize the reproductive, neurodevelopmental, and genetic aspects of HH in human pathology.

## Introduction

**Figure 1:**
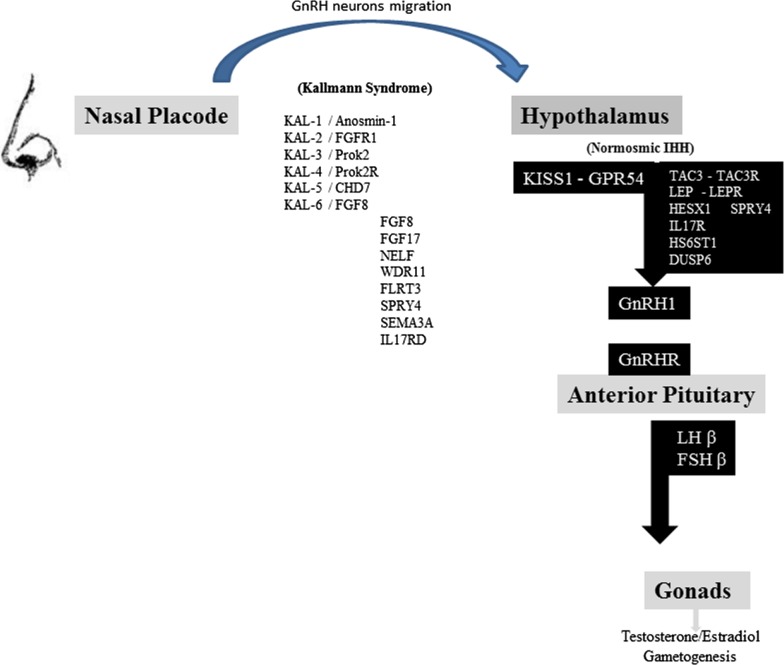
**Schematic representation of the reproductive axis and the different genes invalidating mutations participating in Kallman syndrome and nIHH**. GnRH neurons migrate from nasal placode to hypothalamus in the first weeks of fetal life. Several genes are represented in the left column: their invalidating mutations are responsible for Kallmann syndrome and olfactory dysfunction. Kiss1 neurons integrate different hormonal, metabolic, circadian inputs from other hypothalamic and brain areas and thus stimulate GnRH neurons firing. Several genes are represented in the right black column, including KISS1-GPR54 system, which invalidating mutations lead to normosmic idiopathic hypogonadotropic hypogonadism. GnRH secretion leads to gonadotropins FSH and LH pituitary release. LH and FSH control sex steroids secretion and gametogenesis [adapted from Valdes-Socin et al. (1), with permission].

Reproductive system development and control in mammals is dependent on specific neurons located in the hypothalamus that secrete gonadotropin-releasing hormone-1 (GnRH-1) and control the pituitary–gonadal axis (Figure 1). During embryogenesis, these neurons originate in the nasal placode and migrate into the forebrain along the olfactory-vomeronasal nerves (1–3). Alterations in this migratory process lead to defective GnRH-1 secretion, resulting in heterogeneous genetic disorders such as idiopathic hypogonadotropic hypogonadism (IHH), and other reproductive diseases characterized by the reduction in or failure of sexual maturation and competence. Another consequence of these migratory neuronal defects can be olfactory dysfunction. Depending of the affected genes, other neurological developmental disorders can also be encountered (1–4).

Thus, idiopathic hypogonadotropic hypogonadism (IHH) is a genetic disease that can occur with a normal sense of smell (normosmic IHH) or in association with anosmia (Kallmann syndrome; KS). To date, mutations in many genes have been described in relations to KS and/or normosmic IHH (nIHH) (Tables 1 and 2). Hypogonadotropic hypogonadism (HH) can also be found in association with other distinctive clinical syndromic conditions, such as Prader Willi syndrome, that are outside the scope of the current review.

**Table 1:** **Genes and phenotype related only with normosmic IHH**.

Genes	Locus	Inheritance	Phenotype	Comment
*GNRH1*	8p21-11.2	Autosomal recessive	Normosmic IHH	Cryptorchidism
*GNRH-R*	4q13.2-3			–
*KISS1*	1q32	Autosomal recessive	Normosmic IHH	–
*KISS1R*	19p13.3			–
*LEP*	7q31.3	Autosomal recessive	Normosmic IHH	Severe obesity
*LEPR*	1p31	
*TAC3*	12q13.3	Autosomal recessive	Normosmic IHH	–
*TACR3*	4q25			–
*DUSP6*	12q21.33	Complex trait	Normosmic IHH	–
*LHB*	19q13.32	Polymorphism and mutations (homozygous and heterozygous)	Normosmic IHH	–
*FSHB*	11p13	Polymorphism and mutations	Normosmic IHH	–

**Table 2:** **Genes, genes product, function, and phenotypes associated to congenital hypogonadism hipogonadotropic with anosmia/hyposmia (KS, Kallmann syndrome)**.

Genes	Locus	Gene product	Function	Inheritance	Type of hypogonadism	Clinical phenotype
*KAL-1 (KS-1)*	Xp22.3	Anosmin-1	Migration of GnRH and olfactory neurons	X-linked	Kallmann syndrome or normosmic IHH	Unilateral renal agenesis, synkinesia
*FGF8 (KS-6)*	10q24	Fibroblast growth factor 8	Migration of GnRH neurons	Autosomal dominant	Kallmann syndrome or normosmic IHH	Cleft lip/relatively common (mid-line defects)
*FGFR1 (KS-2)*	8p11.22	Fibroblast growth factor receptor	Migration of GnRH neurons	Autosomal dominant	Kallmann syndrome or normosmic IHH	
*FGF 17*	8p2.3	Fibroblast growth factor 17	Migration of GnRH neurons	Autosomal recessive	Kallmann syndrome or normosmic IHH	
*FLRT3*	20p12.1	Fibronecting like domain containing leucine enrich transmembrane protein 3	Interaction with FGFR	Complex trait	Kallmann syndrome	FGF network
						KO mouse is embryonic lethal
*DUSP6*	12q21.33	Dual specific inhibitor phosphatases	Inhibitor of MAPK pathway	Autosomal recessive	Kallmann syndrome	FGF network
*IL17RD*	3p14.3	Interleukin-17 receptor	Early stage of GnRH specification	Autosomal recessive	Kallmann syndrome	FGF network
*SPRY4*	5q31.3	Sprouty homolog interactor with FGFR1	Inhibitor of MAPK pathway	Autosomal recessive	Kallmann syndrome	FGF network
*CHD7 (KS-5)*	8q12.1-q12.2	Chromatin remodelating factor		Autosomal dominant	Kallmann syndrome or normosmic IHH	CHARGE Syndrome
*SEMA3A*	7q21.11	Semaphorine 3A	Axonal path finding of GnRH neurons	Autosomal dominant	Kallmann syndrome	–
*PROK2 (KS-3)*	3p21.1	Prokineticin-2	Migration of GnRH neurons	Autosomal dominant and recessive	Kallmann syndrome or normosmic IHH	Obesity, epilepsy, sleep disorders, fibrous dysplasia, and synkinesia
*PROKR2 (KS-4)*	20p13	Prok receptor		Kallmann syndrome or normosmic IHH	
*NELF*	9q34.3	Nasal embrionic LHRH factor	Migration of GnRH neurons	Digenic model (in association wth FGFR1 and HS6ST1)	Kallmann syndrome or normosmic IHH	–
*WDR11*	10q	WD repeat containing protein family	Development of neurons	Autosomal dominant	Kallmann syndrome or normosmic IHH	–
*HS6ST1*	2q21	Heparan sulfate 6-O Sulfotransferase	HS modifier	Complex trait	Kallmann syndrome or normosmic IHH	–
			Regulates neural branching	

In this review, we focus on genetic central hypogonadism, which is more frequently encountered in males than in females. Congenital IHH is a clinically and genetically heterogeneous disorder (3, 4). Although sporadic cases predominate, families with congenital IHH have been reported with X-linked, autosomal dominant (AD) or autosomal recessive (AR) inheritance patterns (1–4). In some families, high variability in reproductive and non-reproductive phenotypic features suggests the presence of complex inheritance. In particular, polygenic (digenic or oligogenic) forms and variable forms of transmission can be found in selected cases (6–11). Indeed, further complexity is added by the remarkable observation of reversibility of the phenotype in some cases of genetically determined hypogonadism (12–16).

## The Human Reproductive Axis

Normal human reproduction and sexual characteristics rely on an intact hypothalamic–pituitary–gonadal axis (HPG; Figure 1). Hypogonadism is defined as the insufficient production of sex hormones with or without disturbed gametogenesis. HH results from a dysfunction of the hypothalamic–pituitary axis interfering with control of gonadotropin secretion (1–3, 16).

During life, the activity of the HPG axis has a tri-phasic pattern of “on-off-on.” A first phase of activity occurs from the 16th week of intrauterine life as well as in the period between the 4th and 10th weeks of postnatal life (or “mini-puberty”). Mini-puberty is characterized by an increase in gonadotropin and steroid hormone secretion. Gonadotropins and sex hormones levels rise to a lesser extent than in true puberty. After mini-puberty, the HPG axis is repressed (“off”) until puberty, when the system is reactivated (“on”). HPG axis activity is maintained throughout adult life in men whereas in women, menopause intervenes, and low sex steroids and compensatory high gonadotropin levels are characteristic (1–3).

The immediate postnatal period can be a window of opportunity for pediatricians and neonatologists to diagnose certain forms of HH. The congenital gonadotropin deficiency phenotype is variable and depends on the gender, the magnitude of the deficit, and the specific genetic abnormalities (Figure 1). At the time of puberty, the diagnosis of HH may be suspected due to the absence in the onset of puberty and development of secondary sex characteristics in both sexes. In adulthood, gonadotropin deficiency can be suspected in a woman without breast development or who presents with primary amenorrhea. In adult men, gynecomastia, small testes (<14 mL), penile hypoplasia, and/or oligo-azoospermia raise the clinical suspicion of congenital hypogonadism (1–3).

### Normosmic idiopathic hypogonadotropic hypogonadism

The genetic abnormalities described below are infrequent or rare (see Table 1). In contrast to KS, patients with nIHH have a normal sense of smell and tend not to have other clinical signs. From a biological point of view, sex steroids secretion and gametogenesis are compromised, but to varying degrees. As it would be expected, reproductive phenotypes are more pronounced in subjects in whom the receptor is inactivated as compared to those harboring hormone inactivating mutations.

#### GNRH-1 and GNRHR mutations

Gonadotropin-releasing hormone (GnRH) is encoded by the *GNRH1* gene, which is located on chromosome 8p21-11.2. GNRH-1 mutations are rare and have been described in only two families (17, 18). A single homozygous mutation (c.18-19insA) affecting the peptide precursor preproGnRH was described in a Romanian family (17). It encoded a truncated and biologically inactive peptide in a male patient and his sister, both of whom had delayed puberty and normal sense of smell. The phenotype was reversed by pulsatile GnRH administration. Another homozygous *GNRH1* mutation was identified in a prepubertal boy from Armenia, with cryptorchidism and microphallus (18).

The *GNRHR* gene (locus on chromosome 4q13.2-3) encodes for the GNRH receptor. There is some variability in clinical expression of *GnRHR* mutations that is due to a partial loss of function. *GNRHR* mutations have been described in about 40–50% of familial AR nIHH cases, and in around 17% of sporadic nIHH (1–3).

#### KISS1 and GFPR54 mutations

The gene *KISS1* was described originally as a metastasis suppressor gene but it is a key gene in reproduction. It is localized on chromosome 1q32, encoding a protein called kisspeptin, which is, in turn, processed in four peptides Kp10, Kp13, Kp14, and Kp54. Kisspeptins stimulate GnRH neuronal firing and GnRH secretion, which then triggers an increased release of LH and FSH (Figure 1). The *KISS1R* gene (locus 19p.13.3), a G-protein-coupled receptor, is also known as the *GPR54* gene, and it is the receptor for kisspeptins. *GPR54* mutations can be compound heterozygous or homozygous (19–22). Loss of function mutations in *KISS1* (20) and *GPR54* cause HH in mice and men (19, 21, 22). Moreover, higher serum kisspeptins levels are found in obese hypogonadal men and in central hypogonadism than in controls (23). In patients with *GPR54* mutations, GnRH deficiency can be partial or complete, permanent, or reversible, and can have a congenital or adult onset. Six homozygous inactivating mutations have been described in 19 individuals with nIHH: their LH and FSH secretion was blunted but normal secretion was restored after exogenous GnRH stimulation (19, 21, 22). Kisspeptins are highly expressed in placenta during pregnancy; different patterns of spatiotemporal expression of *KISS1* and *KISSR* were described in normal and pathological placentas (24).

#### TAC3R and TAC3 mutations

The *TACR3* gene (chromosome 4q25) encodes the neurokinin 3 receptor (NK3R) and the *TAC3* gene (chromosome 12q13.3) encodes neurokinin B (NKB), its endogenous ligand. nIHH caused by mutations in *TAC3* and *TAC3R* have an AR heritance (25). As well as the *GPR54/Kisspeptin* system, *TACR3/TAC3* pathway stimulates GnRH neurons. In initial studies, defects in either *TAC3* or *TACR3* were found in 11 patients from 5 of 10 families studied, but in none of 50 sporadic cases (25–27). Francou et al. studied the gonadotropin axis dysfunction associated with nCHH due to TAC3/TACR3 mutations: it was related to a low GnRH pulsatile frequency leading to a low frequency of alpha-subunit pulses and to an elevated FSH/LH ratio (27). They suggested that this ratio might be useful for pre-screening nCHH patients for TAC3/TACR3 mutations. In another broad cohort of normosmic IHH patients, 7 of the 16 males and 5 of the 7 females with *TACR3/TAC3* mutations were assessed after discontinuation of therapy: 6 of the 7 males and 4 of the 5 females demonstrated evidence for reversibility of their hypogonadotropism (14).

#### Leptin (Ob) and leptin receptor mutations

Leptin is an adipocyte secreted protein that ensures a link between body fat and the reproductive axis. HH and severe obesity are seen in humans and ob/ob mice with genetic leptin deficiency. There are at least 12 patients with leptin deficiency and homozygous mutations. In such cases, recombinant leptin administration restores gonadotropin secretion and dramatically reduces body mass index. Defects in the leptin receptor are more common, being identified in 3% of severe early onset obesity patients. Interestingly, the leptin receptor is expressed on kisspeptin neurons whereas leptin administration induces the expression of *Kiss-1* in *ob/ob* mice (3, 28).

#### LHB mutations

The *LHB* subunit gene is located at chromosome 19q13.32. Five mutations have been published up to now; clinical and molecular data are summarized in Table 3. The syndrome of preserved spermatogenesis with androgenic failure (now known to be due to LH deficiency) was described for the first time by Pasqualini and Bur in 1950 (29). The term “fertile eunuch” was then coined to describe these men.

**Table 3:** **Clinical, biological, pathological, and genetic studies in patients with LH deficiency**.

	Weiss et al. (30)	Valdes-Socin et al. (31)	Lofrano-Porto et al. (32)	Achard et al. (33)	Basciani et al. (34)
Mutation LH beta	Glut54Arg	Glyc36Asp	IVS + 1G > C	Del10HisProlLeu	IVS + 1G > C
	Homozygous	Homozygous	Homozygous	Homozygous	12-bp deletion in
					Exon 2
					Heterozygous
Exon localization	Exon 2	Exon 2	Intron 2	Exon 2	Exon 2
LH Functional Studies	Reduced LH bioactivity	Knot cysteine	Abnormal tertiary structure	Reduced LH bioactivity	No LH secretion
		No LH dimerization	No LH dimerization	
Plasma LH	LH = 64	LH undetectable	LH undetectable	No detectable LH	LH undetectable
Women	No	No	1, amenorrhea	1, amenorrhea	1, oligomenorrhea
Men	One man, impuberism	One man, impuberism	Two men, high FSH et SUα	One man, impuberism	One man
	FSH = 113	Hypoandrogenism		FSH = 20.7	FSH = 8.7
		FSH = 23		SUα = 1.28	
		αSU = 0.8		inhB = N	inhB = N
		inhB = N		High AMH	
Testis biopsy	Leydig = 0	Leydig+	Leydig = 0	Leydig±	(after hCG) Leydig+
	Arrested SPG	SPG diminished	Arrested SPG	SPG+	SPG+
Fertility	–	Azoospermia	Azoospermia	Normospermia but abnormal forms.	Oligospermia
Treatment	T2 then hCG	T2 then hCG	T2	T2 then hCG	T2 then hCG

*All but one patient (Basciani et al.) are homozygotes for an inactivating βLH mutation*.

*SU α, alpha subunit; inhB, inhibin B; AMH, antimullerian hormone; SPG, spermatogenesis; T2, testosterone; N, normal; Anorm, abnormal; Dim, dimerization*.

*Normal values: FSH (2–14 UI/L), LH (2–10 UI/L), alpha subunit (<1.2 mUI/L)*.

In affected men, sexual differentiation was normal, but the absence of or significantly reduced LH secretion restrained the induction of puberty and altered Leydig cell proliferation and maturation (30–34). These males have impaired spermatogenesis, ranging from azoospermia to oligospermia, which has been linked to the lack of LH stimulation and low intratesticular testosterone action (5, 30–35). In 2004, we described a man with a homozygous missense mutation (G36D) in the *LHB* subunit gene that abrogated subunit dimerization and rendered LH biologically and immunologically inactive (31). Treatment with human chorionic gonadotropin (hCG) induced near normalization of testicular structure (5). The patient and his wife conceived a child by intracytoplasmic sperm injection from ejaculated sperm. The male heterozygous child had normal LH, FSH, and testosterone levels, at the age of 4 weeks (5, 35).

In women, *LHB* mutations lead to a normal pubertal development but they can have primary amenorrhea and micropolycystic ovaries (32–34).

#### FSHB mutations

The β subunit of FSH (*FSHB*) is located at chromosome 11p13. Three men and four women with inactivating FSH mutations have been reported. Men have normal pubertal development although they have azoospermia, whereas women have abnormal pubertal maturation; in these patients high level of LH are found whereas FSH is low/undetectable. Estrogen and progesterone concentrations are low (1, 2).

#### Gonadotropins receptor (LHR and FSHR) mutations

Inactivating mutations affecting the gonadotropin receptors contrast with those affecting their ligands in that they are invariably associated with hypergonadotropic hypogonadism; hence, they are not discussed here.

### Kallmann syndrome

Kallmann syndrome, involving the characteristic features of HH and anosmia was noted in the historical literature long before being properly characterized as a genetic disorder. A man with delayed puberty and the lack of olfactory bulbs was reported over 150 years ago by the Spanish doctor Aureliano Maestre de San Juan (1828–1890). The German Franz Kallmann (1897–1965) completed in the 1940s a description of hypogonadism and anosmia in two families, establishing the genetic basis of transmission. The Swiss scientist, Georges de Morsier (1894–1982) provided the neuropathological description of the syndrome. KS has a prevalence of 1/5000, with a clear male predominance (1–3, 36, 37). Several mechanisms of inheritance and molecular mutations are described here after and summarized in Table 2.

#### Anosmin-1 (KAL-1) mutations

The *KAL-1* gene is located on the X chromosome at Xp22.3. *KAL-1* encodes anosmin-1, a glycoprotein playing an in important role in kidney, respiratory tract, digestive system, and brain embryogenesis (1–3, 37, 38). Anosmin-1 is an adhesion molecule located on cell surface, consistent with the underlying defect of embryonic neuronal migration in KS. Anosmin-1 is mainly involved in growth and migration of GnRH, mitral olfactory cells, and Purkinje cerebellum neurons. Mutations in *KAL-1* gene cause 14% of familial cases of KS and 11% of cases of sporadic cases (1–3, 38). *KAL-1* mutations lead to HH with or without anosmia and may include synkinesis (mirror movements), unilateral renal aplasia, and mid-line abnormalities such as cleft lip/palate (37, 38).

#### FGF8 (KAL-6), FGF17, and FGFR1 (KAL-2) mutations

The *FGF8* gene (also known as *KAL-6*) is located on chromosome 10q24. Fibroblast growth factors (FGF) interact with FGF tyrosine kinase receptors to mediate growth and development. *FGF8* participates in gastrulation, regionalization of the brain, and organogenesis of the limb and face as an embryonic epithelial factor. *FGF8* and its receptor *FGFR1* are involved in GnRH neuron migration. *FGF8* inactivating mutations can lead to both KS and nIHH with an AD inheritance. Triallelic inheritance has also been described. In addition, cleft lip or palate and other mid-line defects have been described in patients with *FGF8* and *FGFR1* mutations. Other features such as corpus callosum hypoplasia-agenesis or nose, ear, and finger abnormalities are more specific of *FGFR1* defects (37, 39, 40).

*FGF17* is located at chromosome 8p2.3 and *FGF17* has a strong sequence identity with *FGF8*. *FGF17* might be implicated in GnRH neuron biology as an alternative to ligand *FGF8b*. Miraoui et al. have identified FGF17 heterozygous mutations in three patients with congenital HH and anosmia and in another individual. In a sporadic male patient with congenital, HH without anosmia (41).

*FGFR1* is located at 8p11.22-p11.23 and *FGFR1* mutations have been identified in 10% of KS. *FGFR1* related KS has an AD inheritance, associated with incomplete penetrance and interfamilial variability. FGFR1 encodes for type 1 FGF receptor, which is expressed in several embryonic tissues. The activation of the FGF–FGFR complex requires two FGF ligands. FLRT3 (Fibronecting like domain containing leucine enrich transmembrane protein 3) also interacts with FGFR (see Table 2). In addition, the binding of heparin or HS: heparan sulfate proteoglycan (see HS6ST1 gene later) have been shown to be essential for FGF receptor dimerization and function (38). Mice with Fgfr1^−/−^ mutations and patients with loss-of-function mutations in *FGFR1* have defective GnRH neuron migration (31). Thus, loss of function mutations in *FGFR1* which is involved in the development of the face, lead to the abnormal morphogenesis of the olfactory bulb, while specific gain-of function mutations in *FGFR1* cause craniosynostosis (37, 39–41).

#### PROK2 and PROKR2 mutations

*PROK2* (locus 3p21.1) and *PROKR2* (locus 20p13) genes encode for prokineticin-2 and its receptor (42). Prok2 and prokr2 gene knockout mice both have agenesis or hypoplasia of the olfactory bulbs, in association with HH. In this model, there is also abnormal GnRH neuron migration (43). Its heritance can be AD or AR. Mutations in these genes are described in up to 6% of KS and 3% of nIHH (1–3, 42). In pituitary deficits associated with septo-optical dysplasia, McCabe et al. (44), described a patient with a heterozygous L173R mutation in *PROKR2* gene, while its healthy mother is homozygous for this mutation. As some controversy exists on the pathogenic role of some PROKR2 mutations, the prevalence given should be interpreted cautiously. Indeed, digenic mutations are encountered (i.e., *PROKR2* and *KAL1*), while heterozygous patients (i.e., AD transmission) are present in families with healthy relatives presenting the same genotype. There are no reliable accessory pathognomonic features of *PROK2/PROKR2* function loss: patients have been described with obesity, sleep disorder, fibrous dysplasia, epilepsy, and synkinesia (2, 3, 43, 44).

#### NELF mutations

The *NELF* gene is located at chromosome 9q34.3. This gene encodes the nasal embryonic LHRH factor. The *NELF* gene is detected in olfactory sensory cells and GnRH cells during embryonic development. It constitutes a guidance molecule for the olfactory axon and GnRH neurons across the nasal region (45). *NELF* mutations have been described in patients with KS, in association with mutations in *FGFR1* or *HS6ST1*, indicating digenic inheritance. More studies are necessary to confirm a relationship between *NELF* and any reproductive and olfactory disorders (2).

#### WDR11 mutations

The *WDR11* locus is at chromosome 10q26.12 and its heritance is AD. It encodes murine Wdr11 that is expressed in the developing olfactory and GnRH migratory pathway and in the adult hypothalamus. *WDR11* biological function is not well understood: however, Kim et al. identified five different heterozygous mutations in nIHH and KS patients. WDR11 probably also plays an important role in puberty (46).

#### CHD7 mutations

The *CHD7* gene that encodes a chromatin-remodeling factor is located on chromosome 8q12.1. Mutations (AD inheritance) of this gene can cause CHARGE syndrome (Colobomata, Heart Anomalies, Choanal Atresia, Retardation, Genital, and Ear anomalies). *CHD7* was screened in nearly 200 patients: 7 KS and nIHH patients were found, 3 of them with olfactory abnormalities. CHD7 mutations were identified in 6% of KS and 6% of nIHH, respectively (1, 2, 47, 48). Laittinen et al. described in 2012, a KS patient with a truncating CHD7 mutation that underwent a reversal of central hypogonadism after therapy discontinuation (15).

#### HS6ST1 mutations

The *HS6ST1* gene (locus 2q.21) encodes a 6-*O*-sulfation enzyme, which is a member of the heparan sulfate enzyme family. The protein is involved in normal neuronal development and may play a role in limb development. In nematodes, HS 6-*O*-sulfate interacts with anosmin-1 and it is involved in function of FGFR1 and FGF8.

*HS6ST1* shows complex inheritance patterns, not following autosomal or recessive transmission. *HS6ST1* mutations were found in KS patients in combination with mutations affecting the FGFR1 gene. *HS6ST1* mutations were found in patients who had nIHH or variable degrees of olfactory dysfunction (KS) as well as with either normal or abnormal olfactory structures (49).

#### IL17RD, DUSP6, and SPRY4 mutations

The *IL17RD* gene (locus 3p14.3) encodes a membrane protein belonging to the interleukin-17 receptor (*IL-17R*) protein family. In a study with eight patients with congenital hypogonadism all had KS, 7/8 had absent puberty, 6/8 showed congenital hearing loss. One *IL17RD* allelic defect is likely to be insufficient, meaning that additional affected alleles in the same and/or other genes must be present to create the phenotype of KS with hearing loss (41).

*DUSP6* (locus 12q22-q23) encodes a member of the dual specificity protein phosphatase subfamily. They negatively regulate members of the mitogen-activated protein (MAP) kinase superfamily (25) Three patients were described with DUSP6 and *FGFR1* heterozygous mutation; they were hypogonadic, while one had hearing loss and the two others had abnormal speech. DUSP6^−/−^ mice are however viable and fertile (41).

*SPRY4* (locus 5q.31.3) gene encodes a protein (sprouty homolog 4), which is an inhibitor of the receptor-transduced mitogen-activated protein kinase (MAPK) signaling pathway. It is positioned upstream of RAS gene activation and impairs the formation of active GTP-RAS. Diseases associated with SPRY4 include germ cell cancer, and testicular cancer. Miraoui et al. identified four anosmic patients with congenital HH (three females and one male) with heterozygosity for a c.530A-G transition in exon 3 of the SPRY4 gene. Another female patient had a heterozygosity for a c.910G-A transition in exon 3 of the SPRY4 gene. These mutations were not found in 155 controls. One of the patients also had hearing loss and another one had abnormal dentition (41).

#### HESX1 mutations

The *HESX1* gene (locus 3p14.3) encodes a protein that is a transcriptional repressor in the developing forebrain and pituitary gland (27). *HESX1* plays an important role in the temporal and sequential development of the forebrain, hypothalamus, optic nerve, and posterior pituitary (28). Mutations in *HESX1* have also been described in isolated growth hormone deficiency and combined pituitary deficiency (50, 51). *HESX1* mutations have been described in 1.4% of IHH/KS patients (50, 51), but as in *PROKR2* mutations this prevalence should be interpreted cautiously.

#### SEMA3A mutations

The *SEMA3A* gene (7q21.11) encodes the semaphorin 3A protein, which regulates axonal path finding and participates in GnRH migration. Deletions and mutations of the *SEMA3A* gene validate a role for *SEMA3A* in KS. Moreover, SEMA3A knockout mice exhibit GnRH dependent hypogonadism and abnormal olfactory bulb innervation (52).

## Conclusion and Perspectives

Kallmann syndrome and nIHH have the potential to unravel the processes behind normal embryonic development and reproductive neuroendocrine maturation (2). The complex biological path from childhood to the onset of human puberty is still incompletely understood (1–3). The molecular mechanism behind IHH remains unknown in a large number of cases. Over the past decade, the same genetic mutations have been described in associated with both KS and nIHH. Moreover, a significant clinical heterogeneity is seen in isolated GnRH deficiency, which might be explained to some extent by oligogenicity (6, 7, 9). In addition, over 60% of central hypogonadic patients have no identifiable mutations, suggesting that yet more disease loci remain to be discovered (1–3). These unidentified genes warrant an integrated research including clinicians, geneticists, and biological investigators to pursue further understanding of these fascinating cases.

## Conflict of Interest Statement

The authors declare that the research was conducted in the absence of any commercial or financial relationships that could be construed as a potential conflict of interest.
